# Iodine Staining of Glycogen Storage in *Caenorhabditis elegans*

**DOI:** 10.21769/BioProtoc.5745

**Published:** 2026-07-20

**Authors:** Hiba Daghar, Éric Samarut, Alex J. Parker

**Affiliations:** 1 Department of Neuroscience, Université de Montréal, Montréal, QC, Canada; 2 Centre de recherche du centre hospitalier de l’Université de Montréal (CRCHUM), Montréal, QC, Canada

**Keywords:** Iodine staining, Glycogen accumulation, *Caenorhabditis elegans*, Microscopy, Quantification, Metabolic storage, Carbohydrate metabolism

## Abstract

Glycogen is a highly conserved macromolecule across species, and its visualization provides critical insights into both physiological processes and disease states. Existing approaches for glycogen imaging in *Caenorhabditis elegans* rely primarily on traditional microscopy slides, which introduce variability in image acquisition and downstream data analysis, limit throughput, and require substantial hands-on time and technical expertise.

Here, we present a standardized, cost-effective, and high-throughput imaging method that enables efficient visualization and quantification of glycogen in *C. elegans*. Our approach utilizes a custom-designed three-dimensional pad containing two to four chambers, allowing control and experimental samples to be processed simultaneously under identical conditions. Worms are exposed to iodine crystals, ensuring uniform staining while minimizing reagent use and handling variability. Imaging is performed using a simple binocular microscope, and analysis is conducted in Fiji, making the workflow accessible to laboratories with minimal specialized equipment or training.

This method also reduces technical variability, shortens turnaround time, and requires only basic reagents and expertise, making it well-suited for both research and teaching laboratories. Importantly, the platform is readily adaptable to other nematode species and scalable for large-scale genetic or pharmacological screening applications. Together, this workflow minimizes technical variability and provides a robust platform for comparative glycogen analysis in *C. elegans*.

Key features

• Standardized glycogen staining method allows less data variability.

• Provides a quantitative tool to measure glycogen buildup in *C. elegans*.

• Requires a customized pad for imaging.

• Macro is available for batch processing.

## Graphical overview


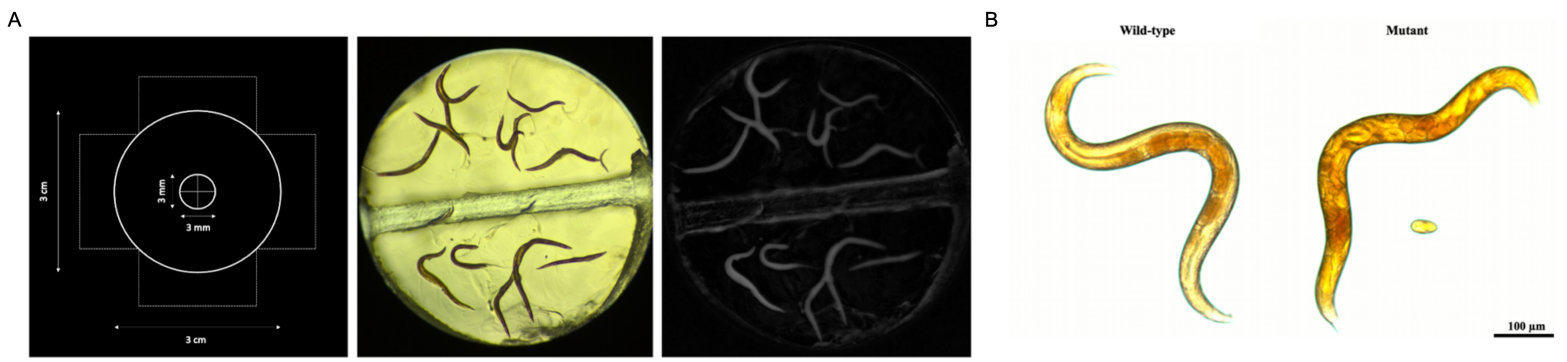


**Overview of the imaging protocol for iodine staining.** (A) From left to right: schematic of 3D-printed pad used for staining, an example of an acquired image after staining, and image processing using a macro in Fiji software for quantification. Control and test worms are simultaneously exposed to iodine. (B) Representative image of glycogen buildup visualized by iodine staining in a COP2008 mutant compared to a wild-type *C. elegans*.

## Background

Iodine-based staining, most performed using Lugol’s solution, has historically been the primary method for visualizing glycogen in *Caenorhabditis elegans*, appearing in light-brown to dark-brown color [1]. However, its quantitative reliability is limited because staining intensity is highly sensitive to iodine concentration and rapidly becomes unstable after application. Early work using Lugol’s solution demonstrated that glycogen localizes to distinct anatomical regions in adult worms, including the area anterior to the posterior pharyngeal bulb, the dorso-rectal ganglion region of the tail, and the most proximal oocytes of the gonad arm [2]. Yet subsequent biochemical studies revealed key limitations: mixtures of polysaccharides produce additive colorimetric signals, and the characteristic red–brown iodine–glycogen complex varies in intensity with glycogen concentration, iodine concentration, temperature, and even the biological source of glycogen. These factors collectively introduce substantial variability into Lugol staining [3,4].

To improve reproducibility, we used unfixed worms, which can be rapidly and effectively stained using iodine vapor. We designed a 3D printed pad to improve reproducibility. The vapor method allows multiple populations to be stained simultaneously under identical exposure conditions. Beyond these technical considerations, glycogen itself plays an important physiological role: worms exposed to a high-glucose diet accumulate roughly twice as much glycogen as controls, and this elevation is associated with a reduction in lifespan, potentially mediated by glucose toxicity, increased reactive oxygen species, or glycogen-dependent signaling pathways [5]. Together, these findings emphasize both the central role of glycogen in *C. elegans* biology and the need for standardized approaches that overcome the inherent variability of traditional staining [3,4].

## Materials and reagents

**Biological materials**

1. *C. elegans* N2 strain [*Caenorhabditis* Genetics Center (CGC)]

2. *C. elegans* COP2008; *agl-1(knu864)* strain (CGC)

*Note: These are the strains presented in this protocol. They can be changed based on the strains of interest.*

**Reagents**

1. Iodine (Sigma-Aldrich, catalog number: 207772-100G)

2. Agarose (Life Technologies, catalog number: 16500-500)

**Laboratory supplies**

1. Erlenmeyer flask (ThermoFisher Scientific, catalog number: 4103-0125)

2. P1000 pipette (Mandel Scientific, catalog number: GF-F167360)

3. P1000 pipette tips (Mettler & Toledo, catalog number: RC-L1000/10)

## Equipment

1. 3D printer (FormLabs, model: Form 3B)

2. Resin (FormLabs, model: Amber Biomed)

3. Leica S6E microscope

4. iPhone 6 (Apple)

5. LabCam Microscope Adapter for iPhone 6/6S Plus (Idu Optics, Directnine)

6. Microscope slides (Cedarlane, catalog number: A547100-5, Fisherbrand^TM^ Premium Clipped-Corner Microscope Slides)

7. Coverslips (Mandel Scientific, NEU-GG-15-PRE, #15mm)

8. Micro-dissecting forceps

9. Scissors

10. Worm pick

## Software and datasets

1. Fiji software (ImageJ2, version: 2.16.0/1.54p, Build: 26d66057dd, Date: 2014-10-15T19:41:44+0000)

2. Prism 9 software (GraphPad, version: 9.0.2)

## Procedure

**A. 3D pad printing**

1. Use the template presented in the Graphical overview to print the 3D pad. Outer circle: 30 mm diameter, 6 mm height. Inner circle: 5 mm diameter and 0.83 mm height.

**Caution:** We used the formal surgical guide resin. Here is some information from the supplier: Surgical guide resin is an autoclavable, biocompatible resin for 3D printing surgical guides, drilling templates, pilot drill guides, and device sizing templates that exceed dental demands in accuracy, part quality, and performance. Surgical guide resin was formulated specifically for FormLabs printers and rigorously tested to meet solvent disinfection and autoclave sterilization standards for implant systems.

**Caution:** The inner chamber dimensions can be adapted based on the diameter of the microscope’s lens. The provided template is suitable for a Leica S6E microscope or equivalent. To adjust the size of the inner chamber, a ruler can be used under the objective lens to measure the required diameter. The inner chamber can be separated into 2–4 sub-chambers to simultaneously test different conditions.

**Caution:** The outer circumference of the pad can be adjusted to match the diameter of the iodine bottle. The provided template is suitable for a 100 g bottle of iodine.

**B. Preparing 3D pads**

*Note: 3D pads should be prepared just before image acquisition.*

1. Place a microscope slide underneath each 3D pad.

2. Cut the end of a P1000 pipette tip.

3. Prepare a 2% agarose solution.

4. Using a P1000 pipette with a cut tip, pipette and transfer the agarose into the chambers of the 3D pad.

5. Using micro-dissection forceps, place a coverslip on top of the poured agarose.

**Caution:** Coverslips should be put directly on the agarose as it solidifies quickly (3–45 s).

**Critical:** Avoid air bubbles when pouring agarose into the chambers.

**C. Image acquisition**

*Note: All these steps require the microscope slide to stay underneath the pad. Any other high-resolution smartphone can be used for acquisition.*

1. Place the iPhone and its adapter on one of the microscope objectives.

2. Place the prepared 3D pad under a binocular microscope.

3. Remove the coverslip with the forceps.

4. Synchronize the *C. elegans* strains of interest and maintain them in standard conditions [6].

5. Transfer 15–20 worms per chamber using a worm pick.

**Caution:** This step might be challenging and requires practice, as worms may stick to the pick and not go onto the agarose. To help with the transfer, a second pick can be used to slide worms on the pad.

**Figure 1.**
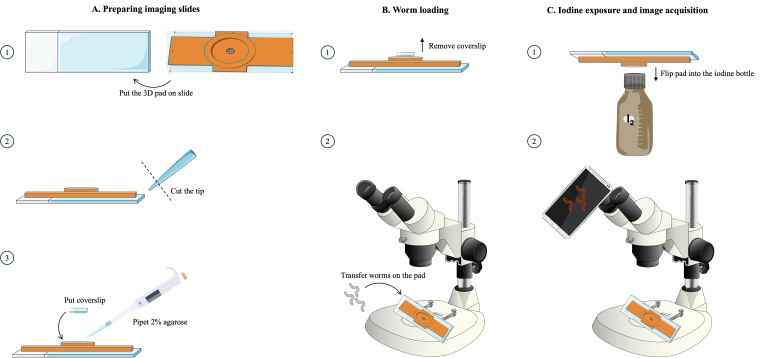
Visual representation of the iodine staining steps

6. Open the iodine bottle and flip the pad into it (see Figure 1C).

**Critical:** The diameter of the pad should fit in the lid of the bottle. In this case, the diameter is 3 cm.

7. Wait 45–60 s.

8. Place the 3D pad back on the microscope and capture the image using the iPhone.

**D. Image processing**

1. Export images in a folder (format .tiff).

2. Open Fiji and use the following macro to process the images:

run("Invert"); run("RGB Stack"); run("8-bit"); run("Stack to Images"); run("Subtract Background"," OK");

3. Select individual worms and measure the integrated density (*Analyze* → *Measure* → *Integrated density*).

4. Normalize the “integrated density” of the test worm on the control worms.

5. Perform the appropriate statistical analysis using Prism software. In this example, where we compare two groups, we performed a Welch t-test. We recommend using a minimum of 15 worms per replicate.

## Data analysis

Data analysis requires the “integrated density” of processed worms (section D). The percentage of staining can be obtained by normalizing test worms to controls. One-way ANOVA or t-test nonparametric analysis can be performed, depending on the sample groups. Further details can be found in [7,8].

## Validation of protocol

**Figure 2.**
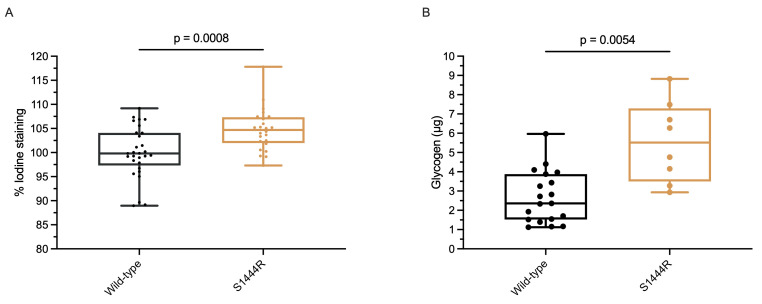
Comparison between iodine staining and quantitative measure of glycogen. (A) Iodine staining quantification shows significantly increasing glycogen buildup in *agl-1* mutant (S1444R) compared to the wild type at day 1 of adulthood (n ≈ 30). (B) Glycogen quantification using a colorimetric kit shows significantly increased levels of glycogen in mutant compared to wild type at day 1 of adulthood. In both plots, each dot represents 1 pellet of 20 mg of worms (N = 3). Data are shown as mean ± SEM. Welch t-test was performed for both tests: (A) t = 3; df = 51.00; (B) t = 3.556; df = 51.00.

In Figure 2, we show that the iodine staining technique replicates the quantitative measure of glycogen when using the gold standard colorimetric kit [Glycogen Assay Kit II (Colorimetric), Abcam, catalog number: AB169558]. Significant glycogen buildup is measured in the *agl-1* mutant compared to the wild type (Figure 2). We were also able to show the accuracy of this method in conditions in which glycogen storage is decreased [7,8].

This protocol (or parts of it) has been used and validated in the following research article(s):

• Daghar et al. [7]. CHK1 inhibition rescues abnormal glycogen buildup in a *Caenorhabditis elegans* model for glycogen storage disease III. *Commun Biol*. (Figure 1C–F). https://doi.org/10.1038/s42003-026-09535-9

• Daghar et al. [8]. Repurposing the HMG-CoA Reductase Inhibitor Atorvastatin for SRD5A3-CDG. *bioRxiv*: 2026 (Figure 3G). https://doi.org/10.64898/2026.01.18.699766
